# Third Examination of Mrs. Cumming by the Commissioner. January 23rd

**Published:** 1852-04-01

**Authors:** 


### THIRD EXAMINATION OF MRS. CUMMING BY THE COMMISSIONER.

### January 23rd.

The Commissioner.—Do you know the amount of your mortgages; can you tell us what mortgages you have on this house and the adjoining house? A. There is a mortgage on each — Q. Do you know the amount of the mortgage on each? A. Why I pretty well know. — Q. Can you tell me what the amount is? A. Is it necessary that I should? — Q. I do not tell you that it is necessary, but it is right and proper that you should tell us; these gentlemen are come, not as your enemies, but as your friends; you will believe that? A. Certainly, for politeness' sake I will believe everything you say. — Q. Can you tell us the amount of each mortgage? A. I think it is a mortgage for 600*l.* — Q. Each? A. But I did not pay the amount; I had not the money then to pay it. — Q. But do you know the amount of the mortgage on each house? (Interruption.) Q. You have sold some of your property to the Railway Company? A. Yes; I know. — Q. Do you know what has become of the money at all? A. It has been invested, some of it. — Q. In the funds? A. In the funds. — Q. In your own name? A. Oh, I never had it in anybody else's name! — Q. You might have it in the name of trustees, you know? A. No; I never had trustees yet. — Q. What is the amount that has been invested, do you know; can you tell me the amount? A. I cannot; 6000*l.* or 7000*l.* — Q. 6000*l.* or 7000*l.* you have invested, have you? A. Yes. — Q. Then why did you not pay off the mortgage on these two houses? A. Because I wanted some of the money to live upon. — Q. Do you not live on your income? A. Yes, but I have a great deal more to lay out than the income from my house; the law suits were very heavy. — Q. What sum do you think you have invested in the funds? I will take care that nobody shall interfere with you. What sum do you think you have invested? You cannot make it out? A. I don't say that. — Q. Don't let me misunderstand you. You do not recollect how much you laid out in law, do you? The law, I am afraid, is desperate, is it not? A. I think it is. — Q. Do you know what you have paid for law expenses? A. Yes, I know what I have paid, but I do not know what I shall have to pay when this is over. — Q. Do you know how much you have paid? A. To defend myself when I was taken to the Horns? — Q. Yes? A. Between two and three thousand pounds. — Q. Who was that paid to? To Mr. Robert Haynes, or to Messrs. Carlon and Haynes? A. Yes, Carlon and Haynes. — Q. Or Robinson and Haynes? A. No, not Robinson and Haynes; Carlon and Haynes. — Q. Do you think you have paid them so much as that? A. Yes, I think so. — Q. They were not your solicitors at the

Horns, you know, were they? A. Robert Haynes came forward there. — Q. Do you think you paid Carlon and Haynes as much as two or three thousand pounds? A. For law? — Q. What law proceedings have they done for you? A. They took that up before—when I was molested before; Carlon and Haynes did. — Q. What, at the Horns tavern? A. The Horns tavern when you presided there. — Q. We understand that your furniture was taken to Oxford-terrace, the other side of the river? A. Very likely it was. — Q. Do you know why it was taken there? A. Because I was so molested at Sir Matthew Wyatt's house, that they gave me no peace at all there. — Q. Who molested you there? I thought it was a quiet, respectable neighbourhood? A. So the neighbourhood is. — Q. Who tormented you there? A. Mrs. Ince, Mr. Ince, and Mrs. Hooper and Mr. Hooper. — Q. Anybody else? A. I think that was enough. — Q. Do you know what rent you were to pay for the place in Oxford-terrace, where you put your things? A. No. — Q. What were you to pay there? A. They are not there now. — Q. How long did they remain there? A. A few months. — Q. Whose house was it? A. I don't know. — Q. Don't you know whose house it was? A. I know pretty well they could not have gone to a stranger's house; it was Mr. Oldfield's house. — Q. In Oxford-terrace, Mr. Oldfield's house? A. I do not know that it was Oxford-terrace. — Q. Do you remember where your furniture went to when it went from Herbert Villa? A. That was the place. — Q. It went from Herbert Villa: where did it go to? A. It went, I think, to the Oldfields'. — Q. Your furniture did not go to the Oldfields'; I thought you had ready-furnished lodgings there? A. Part furnished; but there were my pictures there. — Q. You took your furniture there? A. A good many things. — Q. Do you know whether your furniture went to Oxford-terrace, on the other side of the river? A. No; I do not know where Oxford-terrace is by the name. — Q. You never lived there? A. Not from my recollection of the name. — Q. Dr. Caldwell has attended you for a long time: have you paid him his fees regularly? A. I paid his fees as it suited me, and I gave him bills for them. — Q. Have those bills been all paid? A. As far as by acknowledgments. — Q. Do you think you owe him anything? A. No, not now, because he has got a note of hand from me—not a note of hand. — Q. A promissory note? A. A promissory note. — Q. Do you know the amount of it? A. Yes, I do. Q. Could you tell these gentlemen? A. Is it requisite. — Q. It is desirable that you should? A. Because he is a medical man. — Q. But it is to see whether it is quite right what they tell us. A. I think it is a very hard case that I should be examined by my children about what I pay to my medical attendants.

(A man servant enters with candles.)

A Juryman (to Mrs. Cumming).—Would you like to have lights? A. It is immaterial to me, sir.

The Commissioner.—Do you know when your banker's book was made up? A. Very lately. — How long ago? A. About two months ago.

A Juryman.—Did you have it regularly? A. Yes, as regularly as I could, in the irregular way in which I was living.

The Commissioner.—Why should you live irregularly? don't they pay your rents from Wales? A. Yes. I am not speaking of my tenants, but when I am hurried and driven about. — Q. Who hurries and drives you about? A. I have repeated two or three times to you, Mr. Commissioner Barlow, that it was Mr. Ince, and Mrs. Ince, and Mrs. Benjamin Hooper. — Q. I was in hopes that you had got rid of that impression? A. I could not get rid of the impression when they were always driving me about. — Q. Where did you see them last to annoy you? A. In this house, at least at the door. — Q. How lately was that? A. A few weeks ago. — Q. Did you see them yourself at the door? A. No. I never go to the door myself. — Q. Who told you that they were there—the servants? A. I saw Mrs. Ince myself come to the door. — Q. Within the last two or three months? A. Oh dear, yes. — Q. Would you mind telling these gentlemen what funds you have? A. Is it necessary?

A Juryman.—It is quite necessary that you should tell us.

The Commissioner.—We want to know whether you are imposed on? A. I am not imposed on, except by my nearest relatives.

A Juryman.—But unless you tell us, we do not know how to act for your benefit. A. I do not know that it would be any benefit to me. If I were cheated, certainly I would communicate it; but Mr. Robert Haynes has always done justice to me.

A Juryman.—At one time you dismissed him, and then went back to him again. A. I did; but then, since that, I have known the reason why he neglected sending me the money.

The COMMISSIONER.—Why did he not send you the money? A. He did not receive it. — Q. During that time, did you employ another solicitor? A. I wanted money, and that was the time that Mr. Thorne was employed to get me money.— Q. Are you quite satisfied Mr. Haynes has no money of yours now in his hands? A. He might have: I do not know. — Q. Has he not 500*l.* or 600*l.* or 1000*l.* in his hands of your money? A. He is not making use of my money; that he is not. — Q. Have you not a mortgage of 3000*l.* on some of your property? A. Yes, there is. — Q. Do you know what that was raised for? A. To pay my debts. — Q. Do you know what you received from Sir Charles Morgan? A. Yes. — Q. What was the sum? A. Between 2000*l.* and 3000*l.* I think. — Q. What did you do with it, do you know? A. I had a great many debts to pay, and I want some money, you know, to live upon. — Q. But you have not been living at a great expense, have you? A. No; but travelling about, you know, causes a great deal of unnecessary expense. — Q. When was it that you were last travelling? A. Why, sir, the last time I was travelling was from Brixton to London. — Q. From Effra Hall? A. Yes. — Q. You cannot tell me what kind of balance you have at your banker's? A. No, not exactly: how can I?

A JURYMAN.—Is your banker's book here now in this house, or at the banker's? A. It is at the banker's.

The COMMISSIONER.—Does Mr. Haynes send you in his accounts half-yearly regularly? A. Yes, he does. — Q. And have you got them all? A. I have no occasion to find fault with him. — Q. But you had occasion at one time? A. That was through a mistake, because he had not got the money; that is, Birch and Davis had not sent the money to him. — Q. Why did you dismiss Mr. Thorne? A. I had enough reason to dismiss him. — Q. What was your reason? no doubt it was a good one: what was it? A. For not keeping his word: he said he would send me money, which he never did. — Q. What money was he to get for you? A. Oh, sir, a few pounds; he got papers out of my hands which he has got now. — Q. You gave them to him, did you not? A. Yes, but not to keep. — Q. Have you asked him to return them? A. I never see him now; I did send a message to him. — Q. Do you remember the poison being found? A. Oh, yes. — Q. I think I may have misled you the other day; there was some in a paper, and something in the milk? A. Yes, those are two distinct things. — Q. But were they at the same time? A. Both at the same time. — Q. What did they tell you was in them? They were analyzed by Dr. Barnes? A. He told me it was in the milk; that the milk was put into a dirty jug by one of the servants, and there was Epsom salts in it. — Q. Could you ever account for their being there? A. No; how could I account for what is going on in my house in the kitchen? — Q. Had you not an impression that your daughters had put it there? A. No; I never said I had. — Q. You never had such an impression? A. I never had.

A JURYMAN.—And you have not that impression now? A. No. — Q. You do not believe that your daughters did it? A. No, I do not. I thought it was very strange indeed. — Q. Have they ever tried to poison you? A. Not to my knowledge. — Q. You do not charge your daughters with an attempt to poison you? A. I do not now. — Q. Why can you not return to your natural affection for them? A. That is not the reason I changed my natural affection; that has nothing to do with it. — Q. Will you allow me, as a friend, to make one suggestion. Dr. Williams says, that when you were at Bassaleg you attended his church; the Rev. Mr. Evans also says you attended his chapel; on those occasions you repeated that beautiful prayer of our Saviour, "Father, forgive us our trespasses as we forgive them that trespass against us;" now, we make it the condition of our asking forgiveness of God, our forgiving others; now how can you reconcile it to your mind to leave the world, and go into the presence of the great Creator, at enmity with your own children? A. I have no enmity towards them. — Q. You had unfortunately a husband, who would not be controlled by you; is it not possible that they may be placed in the same situation. I state this with the greatest affection towards you, to consider the relative position in which they stand, and the position in which we all stand; God has put it, that we are only to ask him for forgiveness of our sins, on the same ground that we are disposed to extend our forgiveness, not only to our children, but to all mankind. (No answer.) — Q. Should you like to see your daughter now? A. No, I should not; I am in very bad health; I am in too ill health to stay here much longer.

A Juryman (to the Commissioner).—I don't understand what Mrs. Cumming said respecting her money in the funds. I think she said she had money in the funds. I wish to know whether she has got the bank receipt which they usually give.

The Commissioner (to Mrs. Cumming.)—How lately have you discovered you were mistaken as to the poisoning? A. After I was set right by the medical men. — Q. That was almost immediately after it had happened? A. Yes; it was a good while after. — Q. But you had such an impression until you were set right. A. No, Sir; I never thought they had put the poison in. — Q. You do not recollect ever stating it to any person that that was your impression. A. No, Sir; never, to the best of my knowledge. — Q. Have you any reason to suspect anybody? do you believe it was done by anybody else? A. No; I was at peace with my neighbours, and I did not think they would do anything of the kind. — Q. Do you recollect your daughter, when you were in the Edgware-road having a bit of dinner with you? A. Never; I was taking my lunch quietly, when Mrs. Ince came and obtruded herself upon me. — Q. Did she not have some with you? Did you not ask Mrs. Ince to come and have a bit with you? Did you not say it was cold, and that you had no one to cut it for you? A. No; that is exaggeration, whoever told you that. — Q. But did she stay all the evening with you? A. No; she did not. — Q. How long did she stay? A. I had no watch to go by. — Q. Was it a short or a long visit? A. A long visit I thought. — Q. She came the next day? A. Did she? — Q. Do you not recollect seeing your daughters? A. I saw them several times. — Q. Where did you see Mrs. Ince last? A. Up at this house.

A Juryman.—Did she threaten to strangle you? A. No, sir; no. — Q. Not there? A. No. — Q. When you were in the Edgware-road? A. I know where you mean.

The Commissioner.—Can you tell us what the amount is that you have invested in the funds? A. It is about 2000*l.*, I think; but really you have all asked me so many questions, and I am extremely ill, that really I am not able to tell.

A Juryman.—Would you allow us to explain that we have come here as friends, and if you have anything to tell us, to inform us on any point, it is all we are asking, for we are endeavouring to do justice to you, and if we can ascertain from you everything you wish to say about your daughters we shall be glad. A. I have nothing to say about my daughters. — Q. Who receives your dividends on your funded property? A. I receive them myself. — Q. Do you go to the Bank to receive them? A. Yes.— Q. When did you go last to the Bank? A. I gave that money to my daughters. — Q. You have nothing in the funds now, then? A. No, I have not. — Q. I thought you said you had 5000*l.* or 6000*l.* in the funds. A. No; they received that money that I might have peace for the remainder of my days, and you see how much peace I have. — Q. Can you still explain this to us? You have mentioned several thousand pounds that you received by the sale of property, and by mortgage, and otherwise; can you give us any idea what has been done with it? A. Yes; I know very well. — Q. That is what we wish to know. A. I have been persecuted so, that I think that had better remain in my own breast till I am dead. — Q. But it allows a suspicion to rest in the minds of other persons which you could remove immediately. We do not wish to take your property from you. A. I do not suppose that as gentlemen you would. — Q. Our only object is to know what has become of the money. You have mentioned several thousand pounds that you have received in one way and the other, and if you could state how it has been applied, whether by paying debts, or investment in the funds, or the purchase of property, so that we could account for it, it would be desirable? (No answer.)

The Foreman.—We only ask it as a test of your accuracy in your accounts, and of your being able to manage your own affairs, that is what we are anxious to obtain, if it is possible, from you? (No answer).

A Juryman.—You receive nothing from the funds now? A. No. — Q. I thought you said you went to receive your own dividends? A. So I did when I had money. — Q. That is many years ago—you have not received any dividends lately? A. No; I never go there for it. — Q. What is your income now, do you think? A. It chiefly consists in landed property. — Q. How much do you receive yearly? A. I do not think I have a right to answer that question. — Q. Just as you please, only it is for our guidance? A. I have no objection to your knowing it, but I think in the present state of affairs I had better keep it to myself. — Q. What is the interest you pay on the mortgage on these houses? A. I pay something upon them, but I have it not in my power to pay it all. — Q. Do you know what interest you pay for the mortgages? (No answer.)

The COMMISSIONER.—Do you know what sum you pay a year for the mortgage? (No answer.) — Q. Do you recollect whom you pay it to? Who receives the rent of the next house? You see there is the 3000*l.* mortgage on your estates in Monmouthshire, and there is a mortgage on these two houses that amounts to a large sum. If you pay the interest on that, what amount of interest do you pay? (No answer.) — Q. Your next-door neighbours—those ladies that live there—have they ever paid you your rent? A. No; I have never demanded it. — Q. Whom do they pay it to? A. Mr. Robert Haynes. — Q. Why should they not come and call and pay it to you—have you ever proposed it? A. No; I have never proposed it to them. — Q. Do you know up to what time it was paid? A. I believe they are very exact in their payments. — Q. When you were in Wales the tenants came to you, and you used to receive the rents yourself? A. Yes. — Q. You gave them your receipts very rightly and properly. Why should you not receive your rents here as well? If persons now owe you money, you ought to receive it. When did you receive any money last from any source—can you recollect? A. From my tenants, do you mean? — Q. No; from any parties. When did anybody give you any money last? A. Mr. Robert Haynes has given me money. — Q. Only Mr. Robert Haynes? A. Yes. — Q. Have you any idea how recently he has given you money? A. Yes. — Q. Does he give you the money himself, or does he pay it into your bankers? A. Sometimes he pays it to my bankers, and sometimes to myself, according as my health is. — Q. Do you recollect how you received the last? A. Yes. — Q. Was it paid to your bankers, or yourself? A. To myself. — Q. Do you remember the amount? A. Yes. — Q. When you draw upon your banker, do you make out your own cheques? A. No; they make them out, and I sign them. — Q. Your friends do? A. Yes. — Q. Have you got your cheque-book? A. No. — Q. You paid Dr. Caldwell, you said, by promissory notes? A. Yes. — Q. They are over-due, and not paid, I suppose? Do you know whether you owe any money to Dr. Caldwell? A. No; I cannot owe him any money now, because I have paid him. — Q. In what way did you pay him? A. By a promissory note. — Q. But that is not very good pay, I am afraid? A. He is satisfied with it. — Q. He never remonstrates with you? A. No; never. — Q. Do you think he is satisfied with those notes? A. Yes. — Q. You sold some property to the Water-works Company, and the Railway Company. Here is a list which I made out of what you sold. Do you remember what you sold to the Railway Company a little while ago? A. In Wales? — Q. Yes. A. To Sir Charles? — Q. No; the Railway. A. Because Sir Charles bought some. — Q. What was the amount that the Railway Company were to give, do you know? A. Mr. Jones, you know, made a bargain with them. — Q. He had nothing to do with the Railway Company? A. As much as he had to do with my property. — Q. He did not take the money, I believe? A. No; he did not take the money. — Q. Can you tell the amount of money which you were to have from the Railway Company? A. Two or three thousand pounds. — Q. What was it from the Water-works Company? A. That was about between two and three thousand pounds. — Q. The Water-works? A. Yes. — Q. That would make between four and five thousand pounds altogether? A. Yes. — Q. There was a Mr. Gething, or some such gentleman? A. Yes; Mr. Gething. — Q. He bought something of you, did he not? A. Yes; he bought sufficient land to build a house upon. — Q. And do you recollect what he gave you—how much did he give you for it? A. He gave about 4000*l.* — Q. Do you remember seeing me at the Horns Tavern, when we were in hopes you would have carried out the agreement which you entered into? A. I recollect you well there. — Q. I did not try to persuade you, did I? A. No.

A JURYMAN.—Do you know Mr. Evans, of Monmouth? A. No. — Q. Evans, whose house you lived in? A. Never in Monmouth. — Q. Newport? A. Newport—yes. — Q. Do you recollect him? A. Yes. — Q. What were you to pay him for the accommodation you were to have there; what terms did you agree upon with him? A. I went there because of the cholera that was raging about Newport. — Q. What were you to pay him? A. I was to pay him about 60*l.* a year. — Q. Did you take it by the year? A. At the rate of that. — Q. Do you recollect whether you made any agreement by the week? A. No; there was no specific agreement, because he was not in the habit of letting; he built it himself. — Q. How many grandchildren have you got? A. I do not know, and therefore I cannot give an account of them. — Q. Do you know what rent your next-door neighbour is to pay you?

Another JURYMAN (interposing).—Do you know how many grandchildren you have? A. I cannot answer you that question, for I have not seen them since two of them died. — Q. Do you know where Mr. and Mrs. Hooper are now? A. No, I do not. — Q. Do you know that she is very ill? A. No, I do not. — Q. Should you not like to see her? A. I am very ill myself. — Q. Have you no desire to see her, being ill? A. No: I should be sorry to hear she was ill. — Q. She is very ill. A. Indeed—I am sorry for it. — Q. Were you not aware of it? A. How could I be aware of it when we do not visit. — Q. Do you not wish to visit? A. No, sir; no. — Q. Would you rather that Robert Haynes had your property than your daughters? A. No; and he never proposed such a thing to me. — Q. Not in that will which you proposed to make? A. That will was done aside. — Q. But you had proposed to give him money; do you recollect that? A. I recollect that perfectly well. — Q. Do you recollect how much you intended to give him? A. Yes: I was very ill at the time.

The Commissioner.—Do you know what it was? A. Two or three hundred pounds, I think it was. — Q. You are sure you did not leave him all your property? A. No, I did not. — Q. That you would not do? A. No, I would not. — Q. Whom did you leave as residuary legatees; who was to have all the residue that you had not given by the other parts of the will? A. Different parties. — Q. Was Mr. Robert Haynes to have the residue? A. I do not recollect it. — Q. Do you recollect signing a will at Mr. Hutchinson's? A. No, I do not. — Q. Are you sure you did not sign one there; when you were very ill, did you not sign many papers which you did not know anything about? A. No, I did not sign any papers at Mr. Hutchinson's; I was too ill. — Q. There was a paper on the 4th of June, or thereabouts, sent to Messrs. Carlon and Haynes about the will; do you remember signing that? A. No, I do not. — Q. To whom did you give instructions about your will? A. To Carlon and Haynes. — Q. Did you not write them a letter first? A. There was a letter written for me. — Q. Who wrote it? A. A very particular friend of mine. — Q. Was it Mr. Robert Haynes? A. No: Mr. Robert Haynes is not a particular friend of mine; he is my solicitor, and has always treated me and behaved to me as a man of honour. — Q. Did he not write instructions for your will to Carlon and Haynes, and did you not sign it? A. It is so many years ago now that I cannot call it to my recollection. — Q. Do you remember what rent the two ladies next door are to give you? A. Yes. — Q. What is it? A. Seventy pounds.

A Juryman.—I wish we could persuade you to be on friendly terms with your daughters? A. If it please God that I live long enough, I will think of my grandchildren. — Q. I thought you did not know how many grandchildren you had? A. No, not if I was on my oath. — Q. Do you recollect your daughters through their solicitor writing a letter, saying they would give up everything themselves if you would think of your grandchildren? A. I recollect something of the kind. — Q. It was a very kind letter? A. I was never entitled to anything but kindness at their hands. — Q. But they are very anxious to be friendly with you? A. They have used me too ill.

The Commissioner.—What is the greatest offence they have committed against you? A. Do you not think it any offence for daughters to persecute their mother, so that I cannot get a moment's peace, not anywhere I go to; I am like a hunted dog—a vagabond.

A Juryman.—You most likely imagine it? A. No, sir; no. — Q. If you had them with you and experienced their kindness, you would not say so? A. Ah, sir! — Q. Has any individual ever tried to prejudice you against your daughters? A. No.

A Juryman.—We should have been so happy to see your daughters and yourself reconciled.

